# Etude comparative des complications liées à l'utilisation du cathéter veineux périphérique avec et sans système clos à bouchon hépariné

**DOI:** 10.11604/pamj.2015.21.302.3142

**Published:** 2015-08-26

**Authors:** Ying Chun Liu, Togo Seydou, Yéna Sadio, Tu Zheng Liang, jin Ge

**Affiliations:** 1 22e Mission médicale chinoise au Mali; 2 Service de Chirurgie Thoracique et Cardiovasculaire, Hôpital du Mali, Bamako, Mali

**Keywords:** Cathéters veineux périphériques, complications, bouchon hépariné, peripheral venous catheters, complications, heparin cap

## Abstract

**Introduction:**

L'utilisation correcte du système clos à bouchon hépariné sur les cathéters périphériques pendant les perfusions est une pratique courante dans les pays développés et aussi dans plusieurs pays en développement selon un consensus international établi. Nous comparons les résultats de la formation de thrombus et de l'infection liées au cathéter veineux périphérique chez les patients ayant bénéficié de perfusion avec système clos à bouchon hépariné (groupe expérimentale) et ceux qui ont été perfusé sans bouchon hépariné (groupe témoin).

**Méthodes:**

Nous avons colligé 100 patients hospitalisés pendant la période de Juillet 2014 à Décembre 2014 dans le service d'hospitalisation de chirurgie thoracique de l'hôpital du Mali qui ont été repartis en 2 groupes de 50 patients chacun pour une analyse comparative. L'observation du thrombus dans la lumière du cathéter est effectuée puis enregistré et tous les cathéters ont été repris pour réalisation de culture bactérienne au laboratoire dans les 2 groupes.

**Résultats:**

Dans le groupe témoin, il existe un thrombus dans la lumière du cathéter dans 36 cas (72%) et l'examen de culture bactérienne était positif dans 90%. Tandis que dans le groupe expérimental on retrouve 3 cas (6%) de thrombose du cathéter et on note une absence de germe dans l'examen bactériologique.

**Conclusion:**

L'utilisation correcte du système clos à bouchon hépariné lors des perfusions peut réduire et prévenir de façon significative les complications liées au cathéter notamment l'occlusion par thrombus, leur migration et la survenue de l'infection.

## Introduction

L'utilisation correcte du système clos à bouchon hépariné sur les cathéters périphériques pendant les perfusions est une pratique courante dans les pays développés et aussi dans plusieurs pays en développement selon un consensus international établi. Le cathéter veineux périphérique est facile à utiliser, confortable pour le patient, permet de réduire le nombre de ponction veineuse lors des soins. Son utilisation est plus avantageuse lors de la prise en charge des urgences. Il est aujourd'hui incontournable dans la pratique médicale des soins infirmiers. Cependant il existe des complications liées à son mauvais usage en milieu de soins telles que l'embolie par la migration des thrombus intra cathéter et la septicémie liée à l'infection du cathéter [1]. Ces complications qui sont graves et multiples peuvent être prévenues par l'utilisation d'un système clos à bouchon hépariné [2]. Le but de cette étude était d’évaluer les résultats de l'utilisation de ce système clos à bouchon hépariné au bout du cathéter veineux dans la prévention des complications pendant les perfusions. Il s'agit d'une étude pilote au Mali.

## Méthodes

**Figure 1:**
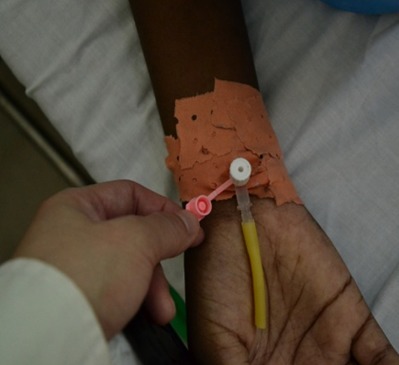
Le cathéter sans système clos à bouchon hépariné utilisé au Mali pour les perfusions avec une fermeture pendant

**Figure 2:**
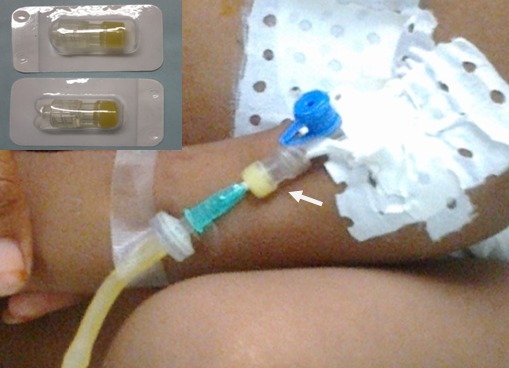
Perfusion avec le système clos à bouchon hépariné

De Juillet à Décembre 2012 nous avons colligé 100 patients dans le service d'hospitalisation de chirurgie thoracique de l'hôpital du Mali. Les patients étaient repartis en deux groupes. Le groupe témoin était composé de 50 patients chez qui le cathéter était simplement utilisé sans système clos à bouchon hépariné (groupe 1) (Figure 1) et le groupe expérimental comprenait 50 patients également avec utilisation du système clos à bouchon hépariné (groupe 2) (Figure 2). L’âge moyen était de 36 ans avec des extrêmes de 2 et 76 ans. Il y avait 45 hommes et 55 femmes. Dans le groupe témoin, après la perfusion la fermeture pendante du cathéter veineux a été utilisée immédiatement pour refermer le cathéter après rinçage. Cette pratique était celle observée au Mali dans tous les services de soins. Dans le groupe expérimental, l'aiguille de perfusion a été placée à travers le système clos à bouchon hépariné mis en place sur le cathéter et après l'arrêt de la perfusion l'aiguille a été retirée tout simplement après rinçage laissant en place le système clos à bouchon hépariné bien fermé empêchant ainsi un contact extérieur direct avec la lumière du cathéter.

**Figure 3:**
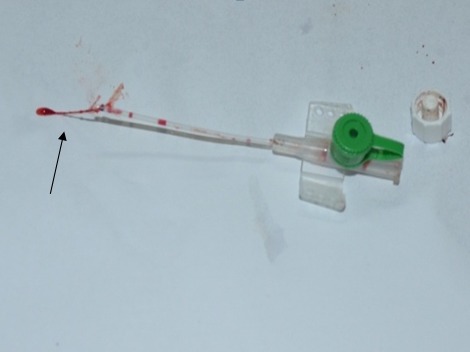
Formation de thrombus après l'ablation du cathéter simple

**Figure 4:**
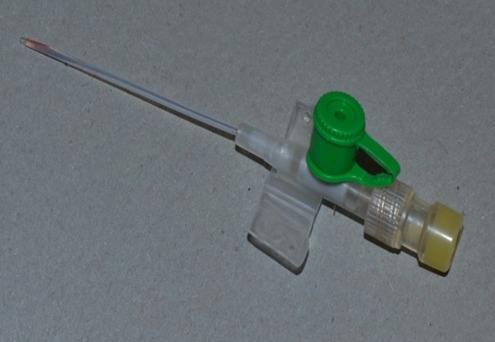
Absence de formation de thrombus après ablation du cathéter avec le bouchon hépariné

Dans les deux groupes de patients la durée de l'utilisation des cathéters était de 3 à 5 jours (moyenne: 4 jours). Les cathéters étaient tous placés au niveau du membre supérieur. Le cathéter a été retiré au bout de 3 à 12 heures (moyenne: 7.5 heures) après l'arrêt des perfusions. La formation de thrombus ou non dans le cathéter chez les patients des 2 groupes a été ensuite vérifiée et enregistrée (Figure 3, Figure 4). Les cathéters ont été tous récupérés dans des conditions d'asepsie rigoureuse pour la bactériologie à la recherche de germe infectieux.

Les complications qui nous semblent être liées à l'utilisation du cathéter veineux observées chez l'ensemble des patients pendant la période d’étude ont été déterminées et également enregistrées. Les données ont été enregistrées puis analysées avec le logistiel SPSS version 10. Les différents résultats ont été ensuite comparés. Le Fisher exact test a été utilisé pour la comparaison dans la formation de thrombus dans les 2 groupes et pour comparer les résultats bactériologiques des deux groupes. La valeur de P < 0.05 a été considéré significatif pour toutes les procédures.

## Résultats

L’âge moyen était de 23 ans (extrêmes: 18 ans et 62 ans). Le sexe masculin était dominant avec un sexe ratio de 1,75. Nous avons retrouvé l'escherichia colli dans 80% des cas du groupe témoin et le pseudomonas aerosinosa dans 10% des cas. La culture était positive que chez 2% des patients du groupe expérimental (Tableau 1). Dans l’étude, 72% des patients chez qui le système clos à bouchon hépariné n'a pas été utilisé ont présenté un thrombus du cathéter et 4% pour les patients du groupe expérimental. Il a été constaté que chez 90% des patients du groupe témoin la culture bactérienne était positive (Tableau 2). Dans le groupe témoin 20% de patients ont présenté des complications parmi lesquelles 10% étaient les thrombophlébites suivies des infections du site (6%) (Tableau 3).

**Tableau 1:** Répartition en fonction de la bactériologie

Bactéries Groupe	Ech. colli	Pseudomonas aerosinosa	Culture positive
Expérimental	1(2%)	0%	1(2%)
Témoin	40(80%)	5(10%)	45(90%)

**Tableau 2:** Tableau de comparaison des 2 groupes par rapport à la formation du thrombus et la bactériologie du cathéter

Groupe	Thrombus formé	pas de thrombus	Culture positive	Culture négative
Expérimental	4 %	96 %	2%	98%
Témoin	72 %	24 %	90%	10%
P Value	P < 0.001	P= 0.005	P < 0.001	P < 0.001

**Tableau 3:** Types de complications rencontrées chez les patients des 2 groupes

Complications	Groupe témoin	Groupe expérimental
Thrombophlébite périphérique	5 (10%)	-
Infection locale du site de perfusion	3 (6%)	-
Infection pulmonaire	1 (2%)	-
Embolie pulmonaire	1 (1 %)	-
Réaction allergique locale	-	1 (2%)
**Total**	**20 %**	**2%**

## Discussion

En France les cathéters veineux insérés par voie périphérique sont posés selon des normes et réglementations qui nécessitent une connaissance de la conduite à tenir face aux différentes complications et des recommandations de bonne pratique concernant une réduction significative des colonisations, des occlusions de cathéter ou thrombose et des protections des emboles [3]. Depuis plusieurs publications dans la littérature, on continue d'observer une augmentation bactérienne après la mise en place de cathéter à cause de la moindre observance de la désinfection du site, de la moindre efficacité de la désinfection, le non respect du rythme de changement et son utilisation pour des prélèvements sanguins [4]. La phlébite est une complication fréquente et souvent grave rencontré dans l'utilisation du cathéter intraveineux. L'incidence est de 2% à 26% [2]. Ceci est comparable aux résultats de notre étude qui a trouvé 10%. Il existe une relation entre la durée de la mise en place du cathéter et l'apparition des phlébites. Aux USA la durée des cathéters est de 3 jours [5], la revue de la littérature en chine rapporte un délai de 5 jours [5, 6].

En général la durée de la mise en place du cathéter de doit pas excéder 3 à 5 jours. Dans notre étude ce délai a été rigoureusement respecté, En dehors de l'effet inflammatoire des médicaments, l'asepsie pendant la mise en place et l'utilisation du cathéter est aussi un facteur primordial de l'apparition des phlébites [7]. La thrombose peut directement obstruer la lumière du cathéter empêchant ainsi de réaliser la perfusion. Ailleurs de petits caillots peuvent se détacher sous pression entraînant souvent des risques d'embolie pulmonaire, de thrombose veineuse profonde et d′autres complications dangereuses [8]. Dans l’étude nous n'avons pas enregistré de cas de thrombose veineuse profonde mais la complication de thrombophlébite périphérique a été la plus observée dans le groupe témoin à cause de l'absence d'utilisation du système clos à bouchon hépariné. Par contre dans le groupe expérimental 4% de patients ont présenté un thrombus de cathéter. Ce résultat obtenu pour le groupe expérimental s'explique par le fait que le cathéter n'a pas été rincé correctement après la perfusion ce qui a entraîne un retour de sang veineux dans le système clos à bouchon hépariné. Lorsque le cathéter est bien rincé son utilisation peut être prolongée avec le système clos à bouchon hépariné et peut également permettre d’éviter les complications. Son mauvais usage peut provoquer une thrombose du cathéter réduisant ainsi son temps d'utilisation [2].

Il est cependant très difficile de rincer correctement le cathéter portant un double orifice avec une fermeture pendante utilisé dans les soins au Mali. Elle comporte 2 orifices avec deux fermetures mobiles. Le retour sanguin dans le cathéter est observé par la différence de pression dans le cathéter et entraîne donc la formation du thrombus. Dans son utilisation multiple il est très facile d'infecter les fermetures aussi bien que les orifices du cathéter entraînant très souvent les phlébites et les infections de site de ponction. La présence de sang dans le cathéter est un milieu de culture qui favorise également l'apparition des phlébites [9] comme nous l'avons observé dans notre étude lors de la bactériologie. Il faut respecter les règles de bonnes pratique, le rythme de changement des lignes, la manipulation des connections, le rinçage du dispositif, placer un nouveau bouchon stérile chaque fois que l'accès ou le robinet a été ouvert, limiter le nombre de réouverture afin de ne pas polluer ou infecter le cathéter [3, 10]. Le système clos à bouchon hépariné est fermé de façon hermétique empêchant ou réduisant la possibilité de pollution du cathéter. Pour les mesures de prévention de la thrombose du cathéter, il faut fermer le régulateur de débit à la fin de la perfusion. Au Total 10 ml de sérum physiologique suffisent pour le rinçage du cathéter. 5 à 6ml de sérum physiologique sont injectés lentement et secondairement 2 à 3 ml rapidement injectés qui seront maintenus dans la lumière du cathéter juste pendant 1 seconde. Il faut répéter cet exercice 2 à 3 fois puis injecter 1 à 2 ml à travers le système clos à bouchon hépariné des qu'il est replacé [1, 7]. Le rinçage correct du cathéter avec bouchon hepariné permet de l'utiliser de façon correcte et efficace.

Lorsque le sérum physiologique est utilisé pour la perfusion, il est recommandé juste avant la fin de la perfusion d'injecter rapidement une bonne quantité de sérum et retirer lentement l'aiguille du système clos à bouchon hépariné [5]. Lorsque la perfusion n'est pas possible, il faut rapidement aspirer dans la lumière du cathéter avec une seringue. Si le bouchon n'est pas perméable, il est préférable de le remplacer. Il ne faut jamais injecter de force ou purger dans le cathéter ce qui pourrait entraîner la migration des caillots et emboles gazeux [1, 5]. Pour les mesures de prévention de l′infection du cathéter, il faut utiliser 2% de povidone iodée pour désinfecter de l′intérieur vers l′extérieur la surface cutané sur environ 8 cm de diamètre. Après séchage de la peau, procéder à la prise de la voie veineuse [11]. Il est recommandé de désinfecter correctement la surface du système clos à bouchon hépariné avec le povidone iodé 2% ou l′alcool à 70° avant chaque utilisation pour la perfusion. Il faut maintenir propre et sec le sparadrap utilisé pour la fixation du cathéter. Nous avons respecté ces normes de bonne pratique chez tous les patients dans le service.

## Conclusion

Le système clos à bouchon hépariné utilisé sur les cathéters périphériques lors de perfusion permet de réduire considérablement les complications liées au cathéter veineux pendant les perfusions. Au Mali, pour des raisons économiques, le cathéter de perfusion est simplement utilisé sans l'association du système clos à bouchon hépariné. Le plus important est le concept de la méconnaissance de son utilisation au sein du personnel infirmier. Pour prévenir les complications liées à l'utilisation des cathéters lors des perfusions, il est urgent d'introduire l'usage du système clos à bouchon hépariné et de renforcer la formation du personnel dans son utilisation.
